# Supplementary material for: The Helicobacter pylori Urease Virulence Factor Is Required for the Induction of Hypoxia-Induced Factor-1α in Gastric Cells
Source: Cancers (Basel). 2019 Jun 10;11(6):799. doi: 10.3390/cancers11060799 (PMC6627347; doi:10.3390/cancers11060799)
Supplement: Supplementary file 1 [file cancers-11-00799-s001.pdf]

cancers

MDPI

Supplementary Materials

The *Helicobacter pylori* Urease Virulence Factor Is Required for the Induction of Hypoxia-Induced Factor-1α in Gastric Cells

Manuel Valenzuela-Valderrama, Paulina Cerda-Opazo, Steffen Backert, María Fernanda González, Nicolás Carrasco-Véliz, Carla Jorquera-Cordero, Sergio Wehinger, Jimena Canales, Denisse Bravo and Andrew F. G. Quest

Mass Spectrometry

The bands that were diminished in *H. pylori* 84-183 *ΔcagA* were excised from gel lanes with extracts from the *H. pylori* 84-183 wt (Figure 2A) and submitted to mass spectrometry analysis, using alpha-Cyano-4-hydroxycinnamic acid as the matrix. Mass spectra data was obtained on a MALDI-TOF Microflex (Bruker Daltonics Inc., Billerica, MA, USA) in positive ion mode by means of reflection detection. For data acquisition the flexControl 3.0 software program (Bruker Daltonik GmbH, Bremen, Germany) was used. Peaks present in the samples were identified using the Mascot PMF tool of mMass. Peptide fingerprint identification by MALDI-MS revealed the presence of the subunits Urease A and Urease B (UreA, partial [*Helicobacter pylori*] and Urease subunit beta [*Helicobacter pylori*]), with scores 135 and 111, respectively. Scores greater than 94, were considered significant ($p < 0.05$).

Table S1. Protein identification. The results of the Mascot Search are shown. Peptides with protein scores greater than 94 were considered significant ($p < 0.05$). The Protein score is −10*Log(P), where P is the probability that the observed match is a random event.

1. ADM51967.1 **Mass:** 15328 **Score:** **135** **Expect:** 4.1×10^{-6} **Matches:** 11
UreA, partial [*Helicobacter pylori*]

ADM51929.1 **Mass:** 15457 **Score:** **135** **Expect:** 4.1×10^{-6} **Matches:** 11
UreA, partial [*Helicobacter pylori*]

ADM51943.1 **Mass:** 15491 **Score:** **135** **Expect:** 4.1×10^{-6} **Matches:** 11
UreA, partial [*Helicobacter pylori*]

ADM51973.1 **Mass:** 15434 **Score:** **135** **Expect:** 4.1×10^{-6} **Matches:** 11
UreA, partial [*Helicobacter pylori*]

ADM51933.1 **Mass:** 14958 **Score:** **121** **Expect:** 0.0001 **Matches:** 10
UreA, partial [*Helicobacter pylori*]

AAR89372.1 **Mass:** 17604 **Score:** **114** **Expect:** 0.00051 **Matches:** 10
UreA, partial [*Helicobacter pylori*]

AAR89370.1 **Mass:** 17798 **Score:** **114** **Expect:** 0.00051 **Matches:** 10

UreA, partial [*Helicobacter pylori*]

AAR89368.1 **Mass:** 17935 **Score:** **113** **Expect:** 0.00064 **Matches:** 10
UreA, partial [*Helicobacter pylori*]

AAM70366.1 **Mass:** 18266 **Score:** **109** **Expect:** 0.0016 **Matches:** 10
urease UreA, partial [*Helicobacter pylori* 26695]

BAX35500.1 **Mass:** 18494 **Score:** **109** **Expect:** 0.0016 **Matches:** 10
urease UreA, partial [uncultured *Helicobacter* sp.]

AAM70409.1 **Mass:** 18863 **Score:** **106** **Expect:** 0.0032 **Matches:** 10
urease UreA, partial [*Helicobacter pylori* SS1]

ADM51959.1 **Mass:** 13518 **Score:** **105** **Expect:** 0.0041 **Matches:** 9
UreA, partial [*Helicobacter pylori*]

ADM51934.1 **Mass:** 13732 **Score:** **105** **Expect:** 0.0041 **Matches:** 9
UreA, partial [*Helicobacter pylori*]

ADM51979.1 **Mass:** 13847 **Score:** **104** **Expect:** 0.0051 **Matches:** 9
UreA, partial [*Helicobacter pylori*]

2. WP_000779240.1 **Mass:** 26636 **Score:** **111** **Expect:** 0.001 **Matches:** 15
urease subunit beta [*Helicobacter pylori*]

WP_000779231.1 **Mass:** 26631 **Score:** **111** **Expect:** 0.001 **Matches:** 15
urease subunit beta [*Helicobacter pylori*]

WP_015427319.1 **Mass:** 26638 **Score:** **111** **Expect:** 0.001 **Matches:** 15
urease subunit alpha [*Helicobacter pylori*]

WP_000779241.1 **Mass:** 26687 **Score:** **111** **Expect:** 0.001 **Matches:** 15
urease subunit beta [*Helicobacter pylori*]

WP_021304234.1 **Mass:** 26661 **Score:** **111** **Expect:** 0.001 **Matches:** 15
urease subunit beta [*Helicobacter pylori*]

WP_021176602.1 **Mass:** 26638 **Score:** **111** **Expect:** 0.001 **Matches:** 15
urease subunit alpha [*Helicobacter pylori*]

WP_024112996.1 **Mass:** 26636 **Score:** **111** **Expect:** 0.001 **Matches:** 15
urease subunit alpha [*Helicobacter pylori*]

WP_025453151.1 **Mass:** 26659 **Score:** **111** **Expect:** 0.001 **Matches:** 15
urease subunit beta [*Helicobacter pylori*]

WP_025454429.1 **Mass:** 26661 **Score:** **111** **Expect:** 0.001 **Matches:** 15
urease subunit beta [*Helicobacter pylori*]

WP_033595251.1 **Mass:** 26647 **Score:** **111** **Expect:** 0.001 **Matches:** 15
urease subunit beta [*Helicobacter pylori*]

WP_033764385.1 **Mass:** 26624 **Score:** **111** **Expect:** 0.001 **Matches:** 15
urease subunit alpha [*Helicobacter pylori*]

WP_000779233.1 **Mass:** 26608 **Score:** **111** **Expect:** 0.001 **Matches:** 15
urease subunit alpha [*Helicobacter pylori*]

WP_050841434.1 **Mass:** 26645 **Score:** **111** **Expect:** 0.001 **Matches:** 15
urease subunit beta [*Helicobacter pylori*]

WP_058905136.1 **Mass:** 26611 **Score:** **111** **Expect:** 0.001 **Matches:** 15
urease subunit beta [*Helicobacter pylori*]

BAW57260.1 **Mass:** 26609 **Score:** **111** **Expect:** 0.001 **Matches:** 15
urease subunit alpha [*Helicobacter pylori*]

WP_077654016.1 **Mass:** 26622 **Score:** **111** **Expect:** 0.001 **Matches:** 15
urease subunit beta [*Helicobacter pylori*]

WP_079333417.1 **Mass:** 26622 **Score:** **111** **Expect:** 0.001 **Matches:** 15
urease subunit beta [*Helicobacter pylori*]

WP_079360426.1 **Mass:** 26638 **Score:** **111** **Expect:** 0.001 **Matches:** 15
urease subunit beta [*Helicobacter pylori*]

WP_079361367.1 **Mass:** 26609 **Score:** **111** **Expect:** 0.001 **Matches:** 15
urease subunit beta [*Helicobacter pylori*]

WP_000779234.1 **Mass:** 26607 **Score:** **111** **Expect:** 0.001 **Matches:** 15
urease subunit alpha [*Helicobacter pylori*]

WP_000779235.1 **Mass:** 26636 **Score:** **111** **Expect:** 0.001 **Matches:** 15
urease subunit beta [*Helicobacter pylori*]

WP_000779232.1 **Mass:** 26722 **Score:** **111** **Expect:** 0.001 **Matches:** 15
urease subunit beta [*Helicobacter pylori*]

WP_001952719.1 **Mass:** 26598 **Score:** **111** **Expect:** 0.001 **Matches:** 15
urease subunit beta [*Helicobacter pylori*]

WP_052917198.1 **Mass:** 26663 **Score:** **110** **Expect:** 0.0013 **Matches:** 15
urease subunit beta [*Helicobacter pylori*]

EMG84461.1 **Mass:** 28167 **Score:** **107** **Expect:** 0.0026 **Matches:** 15
urease, gamma subunit [*Helicobacter pylori* GAM101Biv]

EMH03662.1 **Mass:** 28220 **Score:** **107** **Expect:** 0.0026 **Matches:** 15
urease, beta subunit [*Helicobacter pylori* GAM244Ai]

EMH24096.1 **Mass:** 28190 **Score:** **107** **Expect:** 0.0026 **Matches:** 15
urease, gamma subunit [*Helicobacter pylori* GAM264Ai]

EMG89535.1 **Mass:** 28643 **Score:** **106** **Expect:** 0.0032 **Matches:** 15
urease, gamma subunit [*Helicobacter pylori* GAM201Ai]

WP_033601539.1 **Mass:** 26624 **Score:** **110** **Expect:** 0.0013 **Matches:** 14
urease subunit alpha [*Helicobacter pylori*]

WP_001937431.1 **Mass:** 26638 **Score:** **110** **Expect:** 0.0013 **Matches:** 14
urease subunit alpha [*Helicobacter pylori*]

WP_041050436.1 **Mass:** 26634 **Score:** **110** **Expect:** 0.0013 **Matches:** 14
urease subunit beta [*Helicobacter pylori*]

3. AAK69741.1 **Mass:** 26568 **Score:** **111** **Expect:** 0.001 **Matches:** 14
urease protein UreA [*Helicobacter pylori*]

AAK69737.1 **Mass:** 26569 **Score:** **111** **Expect:** 0.001 **Matches:** 14
urease protein UreA [*Helicobacter pylori*]

AAK69739.1 **Mass:** 26569 **Score:** **111** **Expect:** 0.001 **Matches:** 14

urease protein UreA [*Helicobacter pylori*]

AAK69729.1 **Mass:** 26583 **Score:** **111** **Expect:** 0.001 **Matches:** 14
urease protein UreA [*Helicobacter pylori*]

4. BAW63498.1 **Mass:** 26622 **Score:** **109** **Expect:** 0.0016 **Matches:** 14
urease subunit alpha [*Helicobacter pylori*]

© 2019 by the authors. Licensee MDPI, Basel, Switzerland. This article is an open access article distributed under the terms and conditions of the Creative Commons Attribution (CC BY) license (http://creativecommons.org/licenses/by/4.0/).
